# Dual regulatory effects of medical bovine collagen sponge on macrophages in vitro

**DOI:** 10.1371/journal.pone.0354588

**Published:** 2026-07-27

**Authors:** Rui Wang, Yu Liu, Jingjing Chen, Zhu Meng, Weiye Ren, Wei Huang, Zhaoxu Wang, Qianqian Han

**Affiliations:** 1 Department of Medical Devices, National Institutes for Food and Drug Control, Beijing, China; 2 YanTai University, Shandong Province, China; 3 Wuxi Biot Biology Technology Co., Ltd, Jiangsu Province, China; 4 Department of Emergency, Beijing Stomatological Hospital, Beijing, China; University of the Witwatersrand, SOUTH AFRICA

## Abstract

Medical bovine collagen sponges (MBCS) are extensively utilized in clinical settings for hemostasis and tissue augmentation owing to their superior biocompatibility, biodegradability and hemostatic properties. Nevertheless, their immunomodulatory influence on macrophage function remains largely unexplored. In this study, we systematically evaluated the effects of MBCS on RAW264.7 macrophages through comprehensive in vitro assays, including proliferation analysis, phagocytosis assessment, cytokine expression and angiogenesis evaluation. Our results demonstrate that MBCS significantly enhances macrophage proliferation and phagocytic capacity while potently stimulating VEGF secretion. Furthermore, MBCS-conditioned macrophage supernatants markedly promote endothelial tubule formation in vitro. Notably, MBCS moderately promotes inflammatory responses in resting macrophages, whereas pretreatment with MBCS effectively inhibits lipopolysaccharide (LPS)-induced pro-inflammatory reactions, as reflected by reduced production of TNF‑α, nitric oxide and CD86. These findings reveal the effects of MBCS on macrophages at the cellular level, providing experimental evidence and mechanistic support for its tissue-repairing functions.

## 1. Introduction

The extracellular matrix (ECM) serves as a dynamic scaffold that regulates essential cellular processes, including proliferation, differentiation, apoptosis, and immune responses [1]. Among its key structural components, collagen—a naturally derived biopolymer—exhibits exceptional biocompatibility, biodegradability and mechanical strength, making it a cornerstone in biomedical applications with widespread use in tissue regeneration. [2,3]. Biomaterials containing collagen can significantly improve therapeutic outcomes in reconstructive surgery, organ transplantation and tissue repair, with an increasing number of related products being developed and launched onto the market [4]. However, implantation of such biomaterials inevitably triggers host immune responses, in which controlled activation facilitates material integration, whereas excessive stimulation induces chronic inflammation and implant failure [5,6]. Macrophages are pivotal effector cells in innate immunity, bridging inflammation, infection defense and tissue homeostasis [7].

The crosstalk between biomaterials and macrophages modulates macrophage behavior and polarization, which represents a critical determinant of the in vivo performance of implanted materials. Following implantation, macrophages are rapidly recruited to the implant site, where they undergo polarization toward either pro-inflammatory (M1) or anti-inflammatory (M2) phenotypes depending on the material properties and local tissue microenvironment [5]. Classically activated M1 macrophages exhibit potent microbicidal activity via pro-inflammatory cytokines (TNF-α, IL-6, NO) and enhanced antigen presentation [8,9]. In contrast, alternatively activated M2 macrophages promote tissue repair by secreting anti-inflammatory mediators (IL-1, IL-10, TGF-β1, BMP2) and upregulating arginase-1(Arg-1) [10,11]. Notably, both subsets secrete vascular endothelial growth factor (VEGF), a critical driver of angiogenesis [12]. Macrophage functional plasticity not only determines immune tolerance but also governs tissue repair outcomes. Thus, elucidating the regulatory effects of materials on macrophage behavior is of paramount significance for assessing the safety and efficacy of biomedical products [13].

Macrophage polarization is coordinately regulated by the physicochemical properties of biomaterials (including structure, stiffness, surface characteristics and composition), material functionalization (such as surface coating, drug loading and cytokine delivery), fabrication and structural design, as well as the local inflammatory microenvironment and host immune status [14]. Rational and precise manipulation of these factors enables directed induction of macrophage polarization from pro-inflammatory M1 toward tissue-reparative M2 phenotypes, thereby mitigating foreign body reactions, promoting tissue regeneration, and enhancing implant integration. Traditional studies mostly focus on unidirectional M1-to-M2 macrophage polarization triggered by collagen biomaterials. Emerging evidence indicates that native collagen scaffolds exert time-dependent bidirectional immunomodulation on macrophages: short-term incubation initiates transient M1-dominant proinflammatory responses for early host defense, whereas prolonged incubation suppresses macrophage inflammatory responses and promotes tissue repair and regeneration [14,15]. This unique dual regulatory feature inspires the present study to explore the effects of MBCS on macrophage function.

Medical bovine collagen sponge (MBCS) exhibits substantial potential in ECM remodeling and wound healing. Composed primarily of type I collagen, MBCS interacts with the native ECM and undergoes cell-mediated degradation, remodeling, and metabolic turnover [16]. However, the immunomodulatory mechanisms underlying MBCS, particularly its effects on macrophages, remain largely elusive. Traditional histopathological assessments of implant responses lack molecular mechanism, raising the need for in vitro models to dissect inflammatory regulation [17]. This study investigated the effects of MBCS on macrophage viability, phagocytic activity, cytokine secretion and pro-angiogenic potential. Under both resting and inflammatory conditions, we further examined MBCS-induced changes in macrophage morphology and phenotypic marker expression. Focusing on this bidirectional immunomodulatory property, our findings uncover the dual immunomodulatory roles of MBCS in macrophages, offering mechanistic insights into collagen–macrophage crosstalk and facilitating its clinical translation toward controlled inflammation and augmented tissue regeneration.

## 2. Materials and methods

### 2.1. MBCS extraction and identification

**Fig 1:**
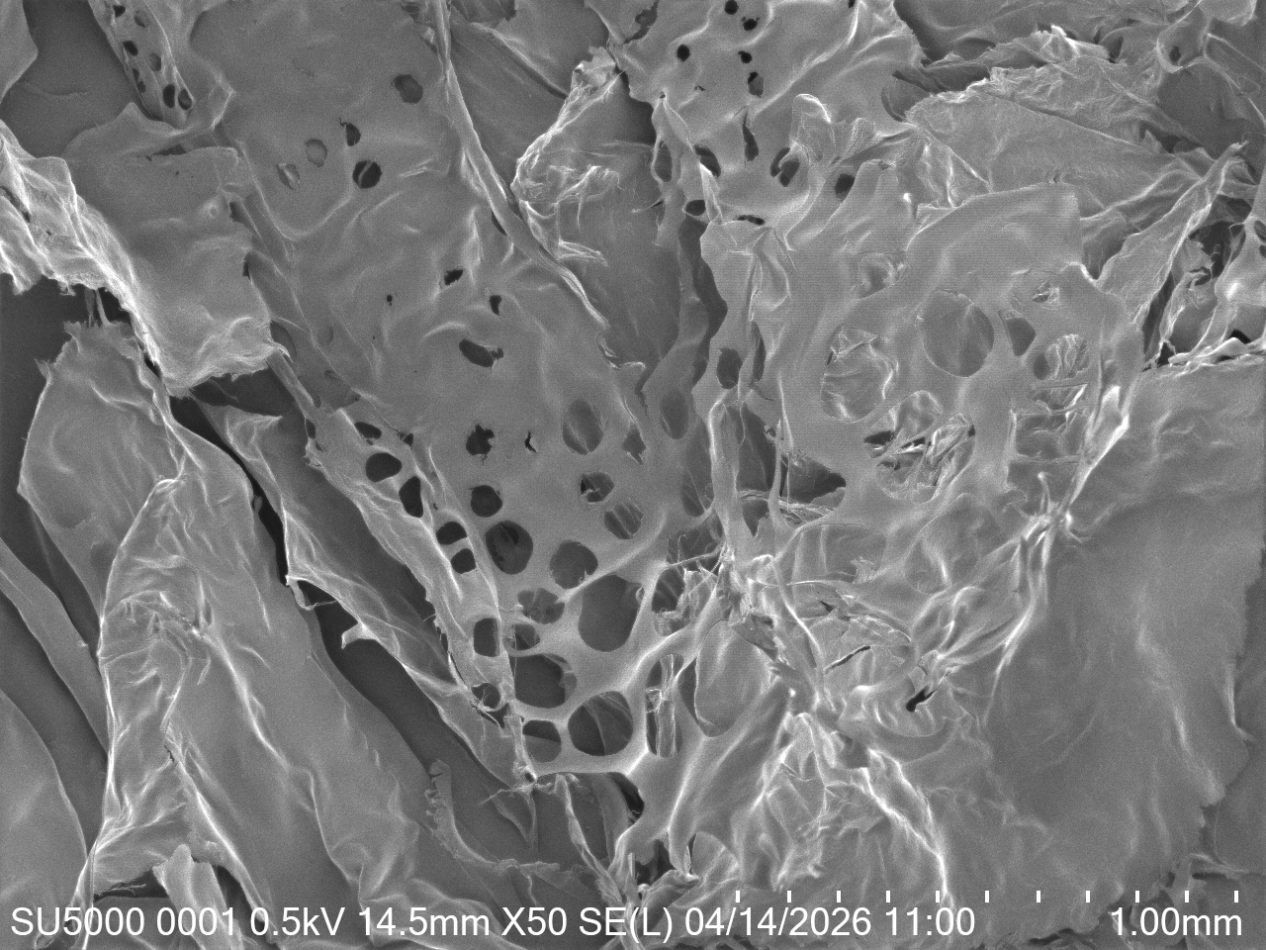
Morphology and microstructure of the MBC used in this study. A. Macroscopic appearance of MBCS. B. Microstructural morphology of MBCS under scanning electron microscopy (SEM).

The raw material for MBCS preparation was derived from bovine Achilles tendon, which underwent acid-enzymatic extraction and purification processes at Wuxi Biot Engineering Co., Ltd (as shown in Fig 1). The extracted collagen was subsequently characterized using multiple analytical techniques: (1) SDS-polyacrylamide gel electrophoresis and Fourier-transform infrared spectroscopy (FTIR) for molecular identification; (2) circular dichroism (CD) and differential scanning calorimetry (DSC) for structural characterization; (3) gel permeation chromatography coupled with low-angle light scattering (GPC-LLS) for determination of molecular weight distribution.

### 2.2. Cell viability and cell cycle

The murine macrophage cell line RAW264.7 was obtained from the Shanghai Cell Bank of the Chinese Academy of Sciences. Cells were maintained in Dulbecco’s Modified Eagle Medium (DMEM; Gibco, America) supplemented with 10% fetal bovine serum (FBS; Gibco, America) and 1% penicillin/streptomycin (Gibco, America) at 37°C in a humidified 5% CO₂ atmosphere. All assays were performed with RAW264.7 cells ranging from P3 to P10, as early passages retain intact macrophage phenotype and stable responsiveness to exogenous material stimulation.

For viability assessment, RAW264.7 cells were seeded in 96-well plates at a density of 1 × 10⁵ cells/mL (100 μL per well) and allowed to adhere overnight. Cells were then treated with varying concentrations of MBCS (0, 25, 50, 100, and 200 μg/mL, fully dissolved in complete medium) and incubated for 6, 24, or 72 h under standard culture conditions. After treatment, 10 μL of Cell Counting Kit-8 (CCK-8; Solarbio, China) was added to each well, followed by 2 h incubation at 37°C in the dark. Absorbance was measured at 450 nm using a microplate reader.

The cell cycle of RAW264.7 macrophages was analyzed using propidium iodide (PI) staining followed by flow cytometry. For cell cycle analysis, RAW264.7 macrophages were seeded in 6-well plates at a density of 5 × 10^5^ cells/mL (1000 μL per well) and cultivated overnight. Following incubation, cells were treated with MBCS at concentrations of 25, 50, 100, or 200 μg/mL and maintained under standard culture conditions (37°C, 5% CO₂) for 24 h. After treatment, cells were harvested through trypsinization, neutralized with complete medium and centrifuged at 350 × g for 5 min. After supernatant removal, cells were washed twice with ice-cold PBS and fixed in 5 mL of chilled 70% ethanol overnight at 4°C. Prior to analysis, fixed cells were pelleted by centrifugation at 500 × g for 5 min and resuspended in 0.5 mL PI staining solution containing RNase A (1 mg/mL). After incubation at 37°C for 20 min in the dark, cell cycle distribution was analyzed using a flow cytometer (BD FACSverse, America), with a minimum of 10,000 events acquired per sample. Data analysis was performed using BD FACSuite software (V1.0.6).

### 2.3. Cell apoptosis

The cell apoptosis of RAW264.7 macrophages was analyzed by Annexin V-FITC/ PI staining followed by flow cytometry. RAW264.7 macrophages in the logarithmic growth phase were seeded in 6-well plates at a density of 5 × 10⁵ cells/mL (1000 μL per well) and allowed to adhere overnight. Cells were then treated with varying concentrations of MBCS (25, 50, 100 and 200 μg/mL) and incubated for 24 h at 37°C in a 5% CO₂ humidified atmosphere. Then cells were harvested using EDTA-free trypsin, washed twice with ice-cold PBS and resuspended in 1 × binding buffer. Subsequently, cells were stained with 5 μL Annexin V-FITC (BD, America) for 15 min at room temperature in the dark, followed by the addition of 5 μg/mL propidium iodide (PI) immediately prior to flow cytometric analysis. Samples were acquired using BD FACSverse within 1 h of staining, with a minimum of 10,000 events recorded per sample. Apoptotic populations were quantified using BD FACSuite software (V1.0.6).

### 2.4. Assessment of macrophage phagocytic activity by neutral red uptake assay

The phagocytic capacity of RAW264.7 macrophages was evaluated using a neutral red uptake assay. RAW264.7 macrophages were seeded in 96-well plates at a density of 1 × 10^5^ cells/mL(100 μL per well) and cultivated overnight. After that, cells were treated with various concentrations of MBCS or lipopolysaccharide (LPS, 1 μg/mL; Sigma-Aldrich, Germany) for 6 h at 37°C in a 5% CO₂ atmosphere. Following treatment, cells were incubated with 100 μL of 0.1% neutral red solution (Sigma-Aldrich, Germany) for 2 h to allow phagocytosis of the dye. Subsequently, the medium containing unincorporated neutral red was carefully aspirated and cells were gently washed twice with pre-warmed PBS to remove extracellular dye. To quantify intracellular neutral red, 100 μL of cell lysis buffer (ethanol:acetic acid = 1:1, v/v) was added to each well. After overnight incubation at room temperature, the absorbance of the lysate was measured at 540 nm using a microplate reader (Molecular device, America).

### 2.5. Cytokine secretion analysis and angiogenesis assay in vitro

RAW264.7 macrophages were seeded in 24-well plates at a density of 2 × 10⁵ cells/ml(500 μL per well) and maintained for 12 h. Cells were then treated with varying concentrations of MBCS (50, 100 and 200 μg/mL) for 72 h. The concentrations of TGF-β1 and VEGF in supernatants were quantified using commercial ELISA kits (TGF-β1 ELISA kit: Elabscience, China; VEGF: ELISA kit: Elabscience, China) according to the manufacturer's protocols. Absorbance was measured at 450 nm using a microplate reader (Molecular device, America).

For angiogenesis experiments, Growth factor-reduced Matrigel (Beyotime Biotechnology, China) was thawed at 4°C and 80 μL/well was added in pre-cooled 96-well plate. HUVECs (obtained from the Shanghai Cell Bank of the Chinese Academy of Sciences) were trypsinized, resuspended in DMEM(high glucose) complete medium and seeded at 2.5 × 10⁴ cells/well. Cells were then treated with varying concentrations of MBCS (50, 100 and 200 μg/mL) for 72 h. Tube formation was assessed using an inverted phase-contrast microscope (Nikon, America) after 3 h incubation at 37°C with 5% CO₂. Three random fields per well were captured and mian parameters (total tube length, branch points) were quantified using ImageJ.

### 2.6. Fluorescent staining

To better observe the effects of MBCS on macrophage morphology under different activation states, we performed fluorescent staining of cellular cytoskeleton and nuclei. RAW264.7 cells were plated in 6-well plates at a density of 5 × 10^5^ cells/well and allowed to adhere completely for 24 hours. Cells were then treated with MBCS(200 μg/mL) for 6 hours. The experiment set two groups: inflammation group and resting group. For the inflammation group, after 6 hours of MBCS treatment, 1 μg/mL lipopolysaccharide (LPS) was added to establish an inflammatory environment, followed by continued culture for 18 hours under the same conditions. The resting group received no additional treatment beyond MBCS exposure and was cultured directly for 24 hours. Following treatment, cells were fixed with 4% paraformaldehyde, permeabilized with 0.5% Triton X-100 and then blocked with 1% BSA at room temperature for 30 minutes. Cells were subsequently stained with phalloidin in the dark for 30 minutes at room temperature, followed by DAPI nuclear staining for 5 minutes in the dark. Finally, samples were examined and imaged using fluorescence microscopy.

### 2.7. Detection of NO, TNF-α and CD86 Expression

The cell culture supernatant collected in section 2.6 was used to measure NO and TNF-α levels. NO concentration was determined using a colorimetric Griess reaction assay (Nitric Oxide Assay Kit, Beyotime, Jiangsu, China). Tumor necrosis factor-α(TNF-α) levels were quantified by ELISA (Elabscience, Wuhan, China) following the manufacturer's instructions.

For CD86 detection, cultured cells from section 2.6 were harvested and analyzed by flow cytometry. Briefly, cells were trypsinized, centrifuged at 500 × g for 5 min and resuspended in 100 μL PBS containing 1 μL anti-CD86 antibody. After 15 min incubation in the dark, cells were washed with 1 mL PBS and centrifuged. The pellet was resuspended in 300 μL PBS and analyzed using a BD FACSVerse flow cytometer (BD Biosciences, USA).

### 2.8. Gene Expression Analysis by RT-qPCR

The expression levels of inducible nitric oxide synthase (iNOS) and TNF-α genes in cells from section 2.6 were analyzed by reverse transcription-quantitative polymerase chain reaction (RT-qPCR). Total RNA was extracted using the RNeasy Mini Kit (QIAGEN, Hilden, Germany) according to the manufacturer's protocol. RNA concentration and purity were determined using a ultramicrofluorometric spectrophotometer (DeNovix Inc., USA). cDNA synthesis was performed with the QuantiTect Reverse Transcription Kit (QIAGEN Gmbh, Germany). Quantitative PCR amplification was carried out using UltraSYBR Mixture (CwBiotech, Beijing, China) on a LightCycler 96 System (Roche, Switzerland). Gene-specific primers (listed in Table 1) were used for amplification and relative gene expression was calculated using the 2-ΔΔCt method with normalization to appropriate housekeeping genes.

**Table 1:** RT-PCR primer sequences.

Primer	Forward (5′-3′)	Reverse (5′-3′)
INOS	GTTCTCAGCCCAACAATACAAGA	GTGGACGGGTCGATGTCAC
TNF-α	CAGGCGGTGCCTATGTCTC	CGATCACCCCGAAGTTCAGTAG
GADPH	AGGTCGGTGTGAACGGATTTG	GGGGTCGTTGATGGCAACA

### 2.9. Statistical analysis

All experiments were performed with at least three independent biological replicates. Data are presented as mean ± standard error of the mean (SEM) unless otherwise specified. Statistical significance was determined using one-way ANOVA or Student's t-test, with significance levels denoted as follows: *p < 0.05, **p < 0.01, ***p < 0.001, and ****p < 0.0001.

## 3. Results

### 3.1. Structural characterization of MBCS

**Fig 2:**
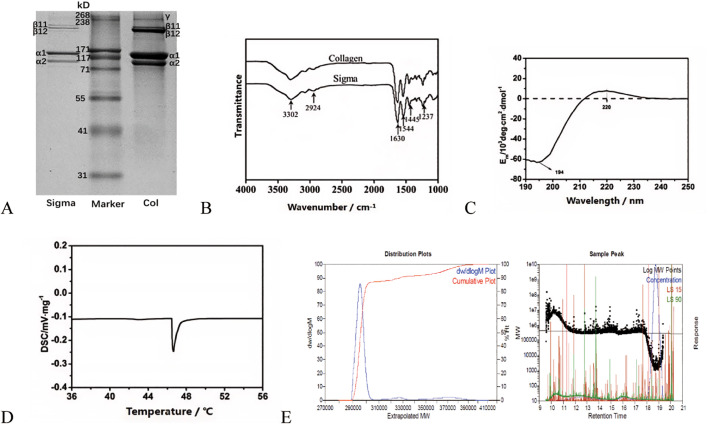
Structural characterization of MBCS. A. SDS-PAGE analysis of MBCS compared with commercial type I collagen control (Sigma). B. FTIR spectra showing characteristic absorption peaks of MBCS and type I collagen control. C. Circular dichroism spectrum of MBCS. D. Differential scanning calorimetry (DSC) thermogram. E. Molecular weight distribution profile: light scattering (LS) signal versus logarithmic molecular weight (Log MW) points.

SDS-polyacrylamide gel electrophoresis revealed characteristic bands corresponding to the α, β, and γ chains of MBCS (Fig 2A), demonstrating preservation of the native triple-helical structure comparable to type I collagen controls. The electrophoretic profile showed: (1) two distinct bands at approximately 110 kDa representing the α1 and α2 chains; (2) higher molecular weight bands >200 kDa corresponding to β12 and β11 dimers; (3) a faint band near 300 kDa indicative of γ chain trimers. Fourier-transform infrared spectroscopy (FTIR) confirmed collagen-specific absorption peaks (Fig 2B). The amide I (C = O stretching), II (N-H bending), and III (C-N stretching) bands collectively verified secondary structure integrity. Notably, the amide I band frequency served as a sensitive indicator of triple-helical conformation, while characteristic peaks at 1445 cm^-1^ and 1237 cm^-1^ further confirmed structural homology with type I collagen controls.

Circular dichroism (CD) spectroscopy demonstrated the signature profile of MBCS (Fig 2C), with a negative peak at 194 nm and positive peak at 220 nm – diagnostic of preserved triple-helical geometry. Thermal stability analysis by differential scanning calorimetry (DSC) revealed a thermal denaturation temperature (Ts) of 46.6°C (Fig 2D), consistent with functional triple-helical structure. Molecular weight distribution analysis yielded a weight-average molecular weight (Mw) of 302,738 Da with low dispersity (Đ = 1.0) (Fig 2E), approximating theoretical collagen values (300 kDa) and indicating predominantly monomeric, homogeneous collagen fractions.

Collectively, these analyses conclusively demonstrate that MBCS maintains the structural hallmarks of native type I collagen, including: (1) characteristic chain composition, (2) triple-helical conformation, (3) thermostable architecture, (4) appropriate molecular weight distribution.

### 3.2. MBCS enhances proliferation and suppresses apoptosis in Raw264.7 cells

**Fig 3:**
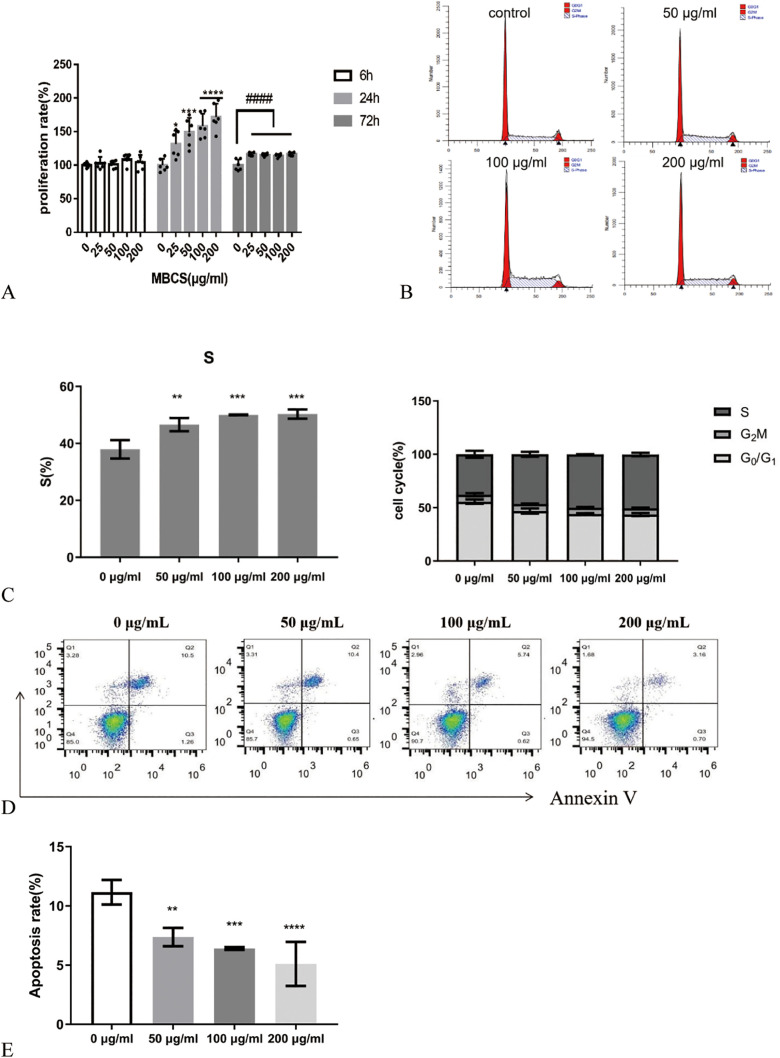
MBCS enhances RAW264.7 cell proliferation by promoting S-phase entry and inhibiting apoptosis. A. CCK-8 assay demonstrating concentration- and time-dependent proliferative effects of MBCS on RAW264.7 cells at 6, 24, and 72 h (n = 6; *p < 0.05, **p < 0.01, ***p < 0.001). B. Representative flow cytometry histograms of cell cycle distribution following PI staining. C. Quantitative analysis of cell cycle phases (G0/G1, S and G2/M) showing significant S-phase accumulation (n = 3), indicative of enhanced proliferative activity. D. Annexin V-FITC/PI dual staining flow cytometry plots illustrating apoptosis inhibition by MBCS treatment (n = 3). E. Statistical analysis of total apoptosis rates (early [annexin V + PI-] and late [annexin V + PI+] apoptotic populations) demonstrating concentration-dependent reduction in apoptosis (n = 3; **p < 0.01, ***p < 0.001).

The CCK-8 assay revealed that MBCS exhibited minimal effects on macrophage proliferation viability after 6 h of treatment (p > 0.05). However, following 24 h exposure, MBCS demonstrated concentration-dependent proliferative effects, with all concentrations showing statistically significant differences compared to the control group (p < 0.05). Notably, 200 μg/mL MBCS increased proliferation viability by 71.8% (Fig 3A). Given the established relationship between cell proliferation, cell cycle progression, and apoptosis [18], we further investigated these aspects. Cell cycle analysis revealed that MBCS treatment for 24 h significantly altered cell cycle distribution in RAW264.7 cells. Flow cytometry analysis (Fig 3B) demonstrated classical G0/G1, S and G2/M phase patterns. Quantitative analysis using ModFit 5.0 (Fig 3C) showed that MBCS treatment resulted in a maximal 21.8% reduction in G0/G1 phase population and a 21% to 32.6% increase in S phase population compared to controls (p < 0.05 for all comparisons). These findings, consistent with the CCK-8 results, suggest that MBCS promotes macrophage proliferation by enhancing S phase entry.

Apoptosis analysis using Annexin V-FITC/PI dual staining demonstrated that MBCS significantly reduced apoptosis in a concentration-dependent manner (Fig 3D-E). Flow cytometry quantification revealed apoptosis rates of (11.15 ± 1.03)% in control cells, which decreased to (7.36 ± 0.77)%, (6.40 ± 0.11)% and (5.10 ± 1.86)% following treatment with 50, 100, and 200 μg/mL MBCS, respectively (p < 0.05 versus control). These results indicate that MBCS not only promotes proliferation but also inhibits apoptosis in RAW264.7 macrophages.

### 3.3. MBCS enhances phagocytic capacity and promotes angiogenic potential in Raw264.7 cells

**Fig 4:**
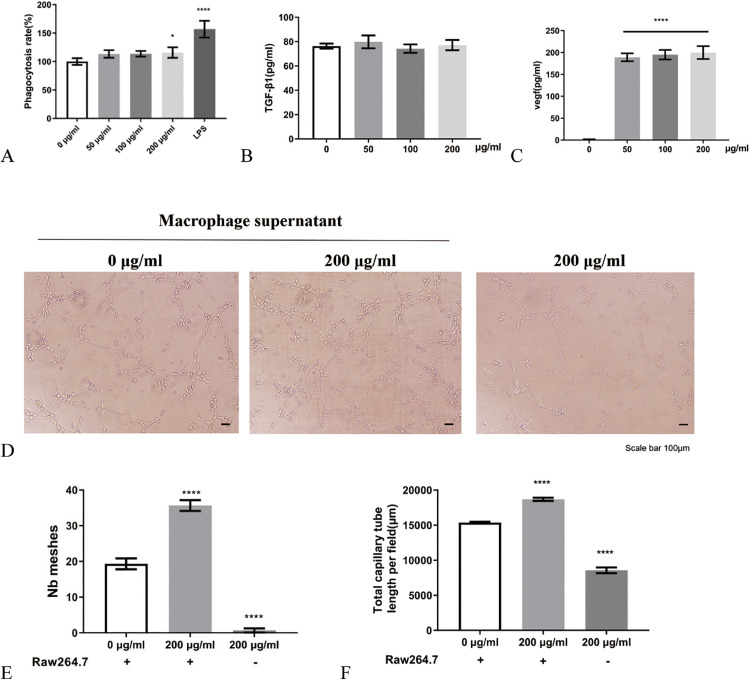
MBCS enhances macrophage phagocytic activity and promotes in vitro angiogenesis. A. Phagocytic capacity of RAW264.7 macrophages following 6 h treatment with MBCS, as determined by neutral red uptake assay (n = 6; *p < 0.05). B-C. Secretion profiles of TGF-β and VEGF from macrophages co-cultured with MBCS for 72 h (***p < 0.001 vs. control). D. Representative images of HUVEC tubule formation after 3 h incubation with conditioned medium from macrophages treated with 200 μg/mL MBCS. E. Quantitative analysis demonstrating significant increases in both tubule length and number in HUVECs cultured with MBCS-treated macrophage supernatant compared to control (n = 3; ****p < 0.0001).

Our findings suggest that MBCS modulates fundamental macrophage functions including cell cycle progression and apoptosis, potentially influencing their primary phagocytic and reparative capacities. The neutral red phagocytosis assay, which quantifies receptor-independent phagocytic activity through cellular uptake of neutral red dye [19], revealed that MBCS significantly enhanced RAW264.7 phagocytosis at the highest concentration (200 μg/mL, p < 0.05) following 6 h treatment (Fig 4A). This early time point was selected based on CCK-8 results showing negligible proliferation effects at 6 h, thereby minimizing potential confounding from cell growth.

Activated macrophages secrete critical mediators of wound repair, including transforming growth factor-β (TGF-β) and vascular endothelial growth factor (VEGF) [20]. While MBCS treatment did not significantly alter TGF-β1 secretion (Fig 4B), it induced dose-dependent increases in VEGF production (Fig 4C). As a central regulator of angiogenesis [21], VEGF facilitates endothelial cell migration and promotes tissue repair [22]. The elevated VEGF levels in MBCS-treated macrophage supernatants prompted investigation of their angiogenic potential using an in vitro tubule formation assay with HUVECs [23,24]. After 3 h incubation, light microscopy demonstrated enhanced tubule formation in cultures supplemented with MBCS-macrophage conditioned medium compared to controls (Fig 4D). Quantitative analysis using ImageJ software revealed significant differences in both tubule number (19.3 vs 35.6 vs 0.67 for control, 200 μg/mL MBCS-macrophage and MBCS-only groups, respectively) and total tubule length (15,374.3 vs 18,689.3 vs 8,569.6 μm for control, 200 μg/mL MBCS-macrophage and MBCS-only groups, respectively) (Fig 4E-F). These results indicate that MBCS-primed macrophages secrete factors capable of stimulating angiogenic responses in endothelial cells.

### 3.4. MBCS modulates morphological and inflammatory responses in RAW264.7 cells under different activation states

**Fig 5:**
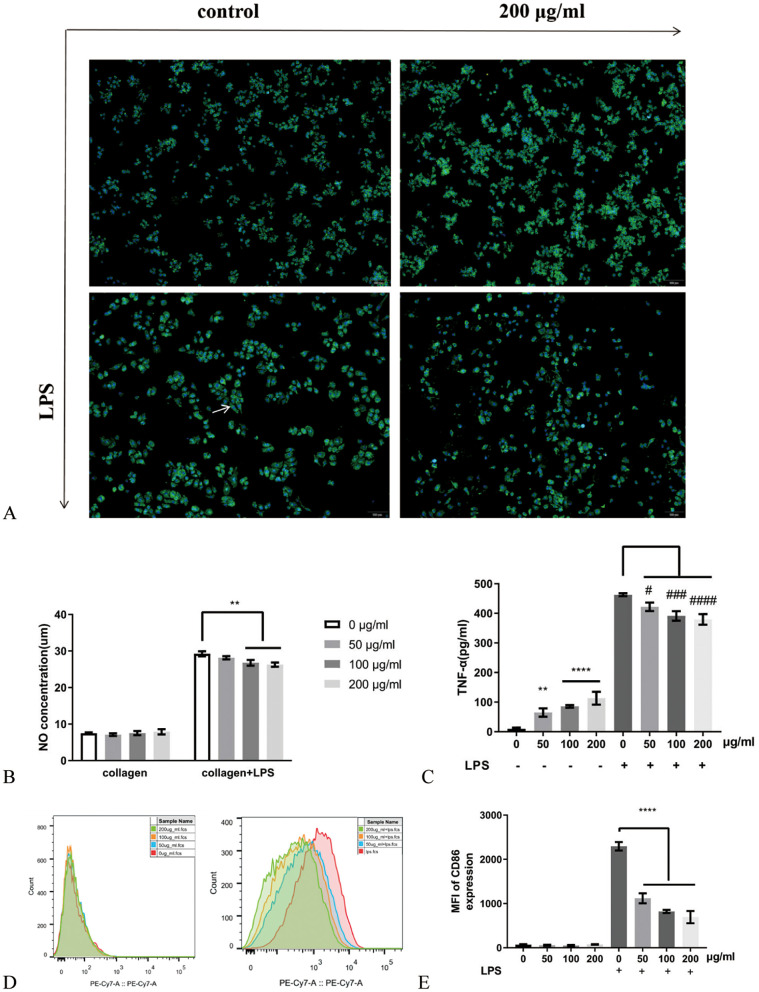
MBCS reduces LPS-induced macrophage activation. A. Representative immunofluorescence images showing DAPI-stained nuclei (blue) and phalloidin-labeled actin cytoskeleton (green). B. Nitrite concentration in culture supernatants, measured at 540 nm, reflecting NO production (p < 0.01 vs. LPS-treated group). C. TNF-α secretion levels following 24 h treatment with LPS or LPS/MBCS combinations (**p < 0.01, ***p < 0.001 vs. control; #p < 0.05, ### p < 0.001, #### p < 0.0001 vs. LPS group). D. Flow cytometry profiles of CD86 surface expression, with fluorescence intensity (x-axis) plotted against cell count (y-axis); rightward shift indicates higher CD86 expression. E. Quantitative analysis of CD86 fluorescence intensity across different groups.

Fluorescence microscopy analysis revealed distinct morphological changes in RAW264.7 cells under various treatment conditions (Fig 5A). Untreated control cells exhibited a typical round shape with abundant protrusions on the surface. Treatment with 200 μg/mL MBCS resulted in a moderate increase in cell density, which was consistent with the previously reported pro‑proliferative effect of MBCS on macrophages, without causing obvious morphological alterations. The production of nitric oxide (NO) was quantitatively evaluated by measuring nitrite levels using the Griess reagent method (Fig 5B). In resting macrophages, MBCS treatment induced a slight concentration‑dependent increase in NO production, but this effect did not reach statistical significance compared with the control group. Meanwhile, the analysis of TNF‑α secretion showed that MBCS promoted TNF‑α release from resting macrophages in a concentration‑ dependent manner, with a statistically significant difference relative to the control group (Fig 5C). Furthermore, flow cytometric analysis of the M1 macrophage marker CD86 revealed that treatment with MBCS alone for 24 h did not significantly up‑regulate CD86 expression (Fig 5D-E). These results indicated that MBCS exerts a mild pro‑inflammatory effect on resting macrophages. A previous study by Guo et al. demonstrated that collapse of the microstructure of collagen sponges can trigger inflammatory responses in macrophages, and the structural stability and cross‑linking type directly determine the intensity of such inflammatory reactions [25]. Specifically, collagen sponges cross‑linked by transglutaminase (TG) underwent microstructure collapse after hydration, leading to elongation and compression of co‑cultured macrophages and inducing significant inflammatory responses. In contrast, collagen sponges cross‑linked by EDC/NHS maintained stable structural integrity after hydration, without affecting macrophage morphology or inducing obvious inflammation, allowing macrophages to maintain a low‑level basal inflammatory phenotype. These findings are consistent with our results, suggesting that collagen sponges maintain a certain baseline level of inflammation in macrophages under resting conditions.

On the other hand, following stimulation with 1 μg/mL LPS, macrophages exhibited a typically activated morphology, characterized by increased cell size and polygonal shape. Notably, pretreatment with MBCS significantly alleviated these morphological alterations and effectively improved macrophage morphology. Furthermore, pretreatment with 100 μg/mL and 200 μg/mL MBCS dose-dependently suppressed LPS-induced NO production (p < 0.01). Consistent with the trend of NO changes, MBCS pretreatment also dose-dependently inhibited the expression levels of TNF-α and CD86. Collectively, these results demonstrate that MBCS pretreatment substantially attenuates the inflammatory activation of macrophages under LPS-induced inflammatory conditions, which may occur through competitively interfering with the binding of LPS to the macrophage surface or related signaling pathways.

### 3.5. MBCS alters mRNA expression of INOS and TNF-α in macrophages under LPS-induced inflammatory conditions

**Fig 6:**
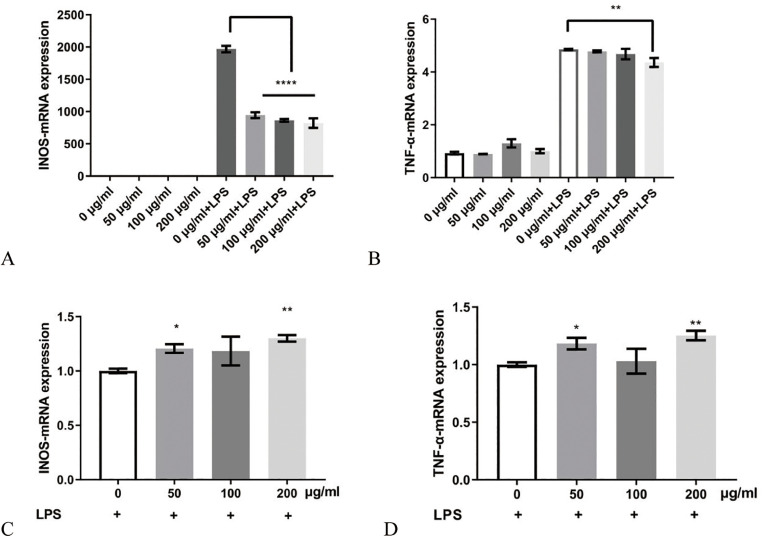
MBCS modulates LPS-induced inflammatory gene expression in macrophages. A, B. RT-qPCR analysis demonstrating that MBCS pretreatment significantly attenuates LPS-stimulated iNOS and TNF-α mRNA expression, while MBCS alone shows no significant effect on these inflammatory markers (n = 3). C, D. Concurrent administration of MBCS with LPS synergistically enhances iNOS and TNF-α mRNA levels compared to LPS treatment alone (n = 3; *p < 0.05, **p < 0.01 vs. LPS group).

To investigate the anti-inflammatory mechanism of MBCS, we quantified mRNA expression levels of inducible nitric oxide synthase (iNOS) and tumor necrosis factor-α (TNF-α) using RT-qPCR. Treatment of macrophages with 50–200 μg/mL MBCS alone caused negligible changes in the mRNA expression levels of these inflammatory markers compared with the control group. In contrast, stimulation with 1 μg/mL LPS resulted in a marked upregulation of TNF-α and iNOS mRNA expression in macrophages (Fig 6A-B). Interestingly, pretreatment with MBCS significantly suppressed LPS-induced iNOS mRNA expression (reduced by 52.0%, 56.2% and 58.3% at 50, 100, and 200 μg/mL MBCS, respectively) and moderately downregulated TNF-α mRNA expression (decreased by 10.3% at 200 μg/mL MBCS). However, simultaneous administration of MBCS and LPS to macrophages led to slight elevations in both iNOS and TNF-α mRNA levels (Fig 6C-D), which were statistically significant at 50 μg/mL (iNOS and TNF-α mRNA increased by 20.6% and 18.3%, respectively) and 200 μg/mL (iNOS and TNF-α mRNA elevated by 30.0% and 25.3%, respectively).

These results indicate that the concurrent exposure of macrophages to LPS and MBCS mildly enhances iNOS and TNF-α mRNA expression at an early stage, rendering macrophages in a slight pro-inflammatory state. In contrast, when macrophages were pre-incubated with MBCS prior to LPS challenge, LPS-triggered inflammatory responses were markedly attenuated. This protective effect may be attributed to the high absorptivity and porous structure of MBCS, which physically adsorb and block the interaction between LPS and macrophages to a certain extent, thereby alleviating the inflammatory response.

## 4. Discussion

Medical bovine collagen sponge (MBCS), as a biocompatible natural scaffold material [26], has been widely applied in wound healing, tissue regeneration and cosmetic augmentation [27–29]. Emerging evidence indicates that MBCS not only provides structural support but also actively participates in tissue repair by modulating immune cell functions [30, 31]. Meanwhile wound healing progresses through four overlapping phases-hemostasis, inflammation, proliferation and remodeling [32]-where macrophage-mediated inflammatory responses play a pivotal role in determining healing outcomes [33]. Macrophages as multifunctional effector cells of the immune system have special meaning in both maintaining tissue homeostasis and executing immune defense [34]. These cells dynamically regulate inflammatory responses through the secretion of pro-inflammatory cytokines (TNF-α, IL-1β, IL-6) or anti-inflammatory factors (IL-10, TGF-β) [35], thereby mediating immunoprotective functions [36]. Furthermore, macrophages contribute to wound healing processes via paracrine signaling of growth factors such as VEGF to promote angiogenesis [37]. Based on these critical functions, we aim to investigate the effects of MBCS on macrophage behavior to elucidate its tissue repair efficacy and underlying mechanisms.

Our investigation reveals that MBCS exerts multifaceted effects on RAW264.7 macrophages, enhancing their phagocytic capacity, promoting proliferation (evidenced by S-phase accumulation and G0/1-phase reduction) and suppressing apoptosis in a dose-dependent manner. Of note, although different types of collagen modulate macrophage phenotypes via distinct signaling pathways—for instance, type VI collagen through the AKT/PKA axis [38] and type II collagen via STAT1 dephosphorylation [39]—MBCS exhibits unique immunomodulatory properties through the bidirectional regulation of inflammatory mediators. Specifically, MBCS mildly upregulates the expression of pro-inflammatory mediators (TNF‑α, NO) in resting macrophages, while markedly suppressing LPS-induced macrophage activation following MBCS pretreatment. This may be explained by the observation that microstructural collapse of MBCS aggravates inflammatory responses in macrophages, whereas MBCS with stable microstructure sustains macrophages in a basally low-inflammatory phenotype. When macrophages are pretreated with MBCS prior to LPS challenge, the porous and adsorptive nature of MBCS may exert anti-inflammatory effects by sequestering LPS and physically blocking its interaction with relevant macrophage surface receptors. Moreover, the pro-healing effects of MBCS extend beyond macrophage modulation to angiogenesis promotion, as evidenced by increased VEGF secretion (though TGF-β levels remained unchanged) and subsequent enhancement of HUVEC tubulogenesis in vitro.

The MBCS employed in this study is clinically indicated mainly for wound hemostasis, tissue filling and wound repair. The present findings demonstrate that preconditioning with MBCS attenuates LPS-triggered inflammatory responses and facilitates tissue repair by promoting angiogenesis. Intriguingly, simultaneous administration of MBCS and LPS slightly upregulated iNOS and TNF-α mRNA expression in macrophages, implying a potential synergistic pro-inflammatory effect. In routine clinical practice, MBCS is predominantly applied in sterile surgical settings to achieve hemostasis in debrided clean wounds. Upon placement on the wound surface, MBCS gradually adsorbs LPS and other inflammatory mediators derived from the wound, which closely mimics the pretreatment regimen in our in vitro experiments. Accordingly, the occurrence of such synergistic pro-inflammatory responses is extremely low under clinical conditions. Nevertheless, if MBCS is applied to severely infected wounds or used in environments with high bacterial loads, it may theoretically carry the potential to mildly exacerbate local inflammatory reactions. Therefore, we recommend that MBCS be preferentially indicated for clean wounds, postoperative hemostasis and repair of non-infected tissue defects to maximize therapeutic benefits and minimize potential risks. Several limitations should be acknowledged in the present study. As our experiments were solely performed in the RAW264.7 macrophage cell line in vitro, this system cannot recapitulate the heterogeneous responses of tissue-resident macrophages in vivo, nor can it replicate the complex regulatory networks of the in vivo microenvironment, including the lymphatic system and systemic circulation. Consequently, the current findings are subject to certain limitations and may not fully reflect the integrated physiological processes in living organisms.

Collectively, this study demonstrates that MBCS exerts beneficial effects on macrophages, including enhanced cellular function and VEGF-mediated angiogenesis, supporting its promise as a candidate biomaterial for wound healing [40]. Furthermore, the divergent regulatory effects of MBCS on macrophages under different experimental conditions provide cellular-level evidence for its pro-repair properties. Our results suggest that timely application of MBCS in the early stages of trauma or on clean wounds may maximize its pro-healing efficacy while mitigating inflammatory risks.

## 5. Conclusion

As a bioactive component, MBCS exhibits dual-directional immunomodulatory effects on RAW264.7 macrophages while simultaneously supporting their fundamental cellular functions including phagocytosis, proliferation and tissue repair. These findings not only enhance our understanding of MBCS-mediated tissue regeneration and its modulatory effects on the local inflammatory microenvironment, but also provide powerful evidence supporting MBCS as a potential tissue repair material, consistent with previous reports [37,40]. The current results may guide future investigations into the precise molecular mechanisms underlying MBCS-mediated immunoregulation.
